# Dosage Compensation in the African Malaria Mosquito *Anopheles gambiae*

**DOI:** 10.1093/gbe/evw004

**Published:** 2016-01-18

**Authors:** Graham Rose, Elzbieta Krzywinska, Jan Kim, Loic Revuelta, Luca Ferretti, Jaroslaw Krzywinski

**Affiliations:** ^1^Vector Molecular Biology Group, The Pirbright Institute, Pirbright, United Kingdom; ^2^Genomics Research Unit, Public Health England, London, United Kingdom; ^3^Centre for Integrative Biology, The Pirbright Institute, Pirbright, United Kingdom

**Keywords:** sex chromosomes, chromosome-wide transcription, RNA-seq, mosquito vectors

## Abstract

Dosage compensation is the fundamental process by which gene expression from the male monosomic X chromosome and from the diploid set of autosomes is equalized. Various molecular mechanisms have evolved in different organisms to achieve this task. In *Drosophila*, genes on the male X chromosome are upregulated to the levels of expression from the two X chromosomes in females. To test whether a similar mechanism is operating in immature stages of *Anopheles* mosquitoes, we analyzed global gene expression in the *Anopheles gambiae* fourth instar larvae and pupae using high-coverage RNA-seq data. In pupae of both sexes, the median expression ratios of X-linked to autosomal genes (X:A) were close to 1.0, and within the ranges of expression ratios between the autosomal pairs, consistent with complete compensation. Gene-by-gene comparisons of expression in males and females revealed mild female bias, likely attributable to a deficit of male-biased X-linked genes. In larvae, male to female ratios of the X chromosome expression levels were more female biased than in pupae, suggesting that compensation may not be complete. No compensation mechanism appears to operate in male germline of early pupae. Confirmation of the existence of dosage compensation in *A. gambiae* lays the foundation for research into the components of dosage compensation machinery in this important vector species.

## Introduction

Sex chromosomes are believed to have originated independently in various groups of organisms from a pair of autosomes after acquisition of a sex determining locus (Bull 1983; Charlesworth 1991). Theory predicts that at early stages of sex chromosome evolution, sexually antagonistic mutations (advantageous to one sex but harmful to the other) accumulate in close linkage to the sex determining locus, which leads to a selection for a reduced recombination within the sex determining region on the proto-Y (Charlesworth et al. 2005). In some organisms recombination suppression spreads to an entire length of the Y chromosome, which, as a result, degenerated through the attrition of genes and accumulation of repetitive sequences to become heterochromatic and nearly devoid of functional genes (Charlesworth 1991; Rice 1994; Bachtrog 2013).

Y chromosome degeneration leaves heterogametic males with a single dose of genes on the X chromosome. The expected 2-fold reduction in transcriptional output compared with the homogametic sex could create deleterious imbalance in genes forming multisubunit complexes or networks spanning the X and the autosomes. Various dosage compensation mechanisms are thought to have evolved to maintain the stoichiometry of the X and the autosomal gene products (Ohno 1967; Veitia et al. 2008; Pessia et al. 2012). In *Drosophila*, the single X chromosome is hypertranscribed in males to achieve a similar expression level to that of autosomes, and to equalize expression between the sexes (Lucchesi 1973). In *Caenorhabditis elegans*, upregulation of transcription from the X chromosomes occurs in both males (X0) and hermaphrodites (XX). It equalizes the expression of X-linked and autosomal genes in males (Deng et al. 2011). A secondary compensatory mechanism, repressing transcription from both X chromosomes by half, protects from deleterious effects of hyperexpression in hermaphrodites (Meneely and Wood 1984; McDonel et al. 2006; Ercan et al. 2007). In mammals, one of the X chromosomes is inactivated in early female embryos, and then the single active X apparently becomes upregulated in both sexes, which results in a balanced expression ratio between the X chromosome and the autosomes (Nguyen and Disteche 2006; Deng et al. 2011; Kharchenko et al. 2011).

Existence of hyperexpression of the active X chromosome in mammals, leading to an expected X:A expression ratio of ∼1.0, was supported by microarray experiments (Gupta et al. 2006; Nguyen and Disteche 2006), but subsequently called into question in the light of RNA-seq data, in which a median X:A ratio was found to be close to 0.5 (Xiong et al. 2010). In the latter study, it was suggested that the earlier analyses might not adequately address the question of dosage compensation because microarrays, being designed for comparing expression of the same genes between different samples, are not sensitive enough to reliably detect small expression differences between genes, as is required for assessing X:A ratios. The above conclusions were contested by several authors who upheld the validity of complete dosage compensation in mammals (Deng et al. 2011; Kharchenko et al. 2011; Lin et al. 2011). Subsequent expression analysis of components of human multisubunit protein complexes indicated that only a small set of X-linked genes are actually dosage sensitive (haploinsufficient) and compensated (Pessia et al. 2012). The debate about dosage compensation in mammals and the lingering uncertainty about the issue (He et al. 2011; Lin et al. 2012; Chen and Zhang 2015) highlight the difficulties in choosing the correct approach to data analysis and interpretation of the dosage compensation status. Although RNA-seq-based approaches are, in principle, free from the limitations of microarrays, with a wider dynamic range and reference-free transcript detection, they can also introduce new biases. Different data processing parameters, in particular at the filtering step, dictating which genes are used for the analysis, may lead to opposing conclusions, even if applied to the same RNA-seq data sets (Kharchenko et al. 2011; Jue et al. 2013). In addition to technical biases, considerations regarding interpretation of the data include potential variation of compensation levels in various tissues and, for insects, in different developmental stages (Jue et al. 2013; Perry et al. 2014).

The African malaria mosquito, *Anopheles gambiae*, has the karyotype consisting of two pairs of autosomes and a pair of fully differentiated sex chromosomes. Males are heterogametic (XY) and have a heterochromatic Y chromosome that seems to have very few active genes (Krzywinski et al. 2004). Dosage compensation in *A. gambiae* was evaluated in a study of microarray data sampled from dissected tissues of male and female adults (Baker and Russell 2011). Approximate parity in the expression levels from the X chromosome and the autosomal genes, indicative of complete compensation, was observed in the ovary and somatic tissues of both sexes. In the testes, expression intensity from the X chromosome was half of the autosomal levels, which could be due to absence of dosage compensation or X inactivation during meiosis (Baker and Russell 2011). A recent study based on RNA-seq analysis of whole-insect samples indicated complete dosage compensation in adults of *Anopheles stephensi* (Jiang et al. 2015), a member of an *Anopheles* lineage that may have diverged from *A. gambiae* lineage 40–70 Ma (Krzywinski et al. 2006; Neafsey et al. 2015).

Sex-biased expression and dosage compensation have been studied in adults of a number of insect species. However, expression in immature stages has been characterized in such a context only in *Drosophila* (Nozawa et al. 2014; Perry et al. 2014). Here we used deep-coverage RNA-seq data to investigate whether dosage compensation exists in the *A. gambiae* preadult stages. Our study included an exhaustive exploration of the effects of filtering genes expressed at low levels on the inference of dosage compensation measures. Transcriptional outputs from the whole-body early fourth instar larvae and pupae were quantified using two approaches, and the median expression levels from the X chromosome and the autosomes were calculated for a range of increasingly restrictive minimum expression thresholds. In pupae, the ratios of the X-linked to autosomal expression were, in general, close to 1.0 (and within the range of chromosome 3 to chromosome 2 expression ratios) in both males and females, consistent with complete dosage compensation. Male to female comparisons of expression at the gene level indicated a slight female bias in the X chromosome expression, likely attributable to a deficit of male-biased X-linked genes. In larvae, the patterns are less clear. The X:A ratios of expression fluctuated in both sexes depending on the expression level, and suggested overexpression from the X chromosome in females when genes expressed at low levels were included in the analyses. Male to female comparisons of the X chromosome expression levels were more female biased than in pupae, suggesting that compensation in larvae may not be complete. The X chromosome appears to be not compensated in the male germline, as the analysis of transcription in the developing testes suggests. We also found that, despite apparent complete compensation in the somatic tissues, expression levels of the candidate dosage-sensitive genes vary considerably during pupal development. Confirmation of the existence of dosage compensation in *Anopheles* lays the foundation for research into the components of dosage compensation machinery in this vector mosquito group.

## Materials and Methods

### Mosquito Samples

Pupae collected for the study originated from the *A. gambiae* Pimperena strain. Larvae used were sampled from progeny of a cross between the Pimperena strain females and males of a transgenic strain with an X-linked fluorescent marker (Magnusson et al. 2012) on a Pimperena genetic background. Mosquito colonies were kept in the insectary conditions at 27 °C, 80% RH, and 12 h light/12 h dark cycle. Adults had constant access to a 10% sucrose solution. Females were fed with time-expired human blood from a blood bank using the Hemotek system. Larvae were reared in pans with deionized water and fed daily with ground fish food (Tetramin). Pans containing fully grown third instar larvae were inspected every 2 h, and newly moulted fourth instar individuals were transferred into marked pans for subsequent sample collection. Similarly, pans with fully grown fourth instar larvae were inspected every hour and newly pupated individuals were transferred into marked pans with clean deionized water. Samples were sexed and then collected at required time intervals. Larvae were sexed under a fluorescence microscope, based on the principle of inheritance of the fluorescently labeled X chromosome by F1 females from transgenic fathers and lack of fluorescent markers in F1 males. Pupae were sexed based on morphological characters.

### RNA Isolation

Samples, each consisting of 13–16 same-sex individuals, were ground in TRIzol and stored at −70 °C until RNA isolation. Total RNA was isolated using PureLink RNA Mini Kit (Ambion) with an on-column DNase treatment step according to the manufacturer’s protocol.

### RNA-Seq Library Generation

RNA quality was evaluated using Qubit RNA assay (Life Technologies) and on a PerkinElmer GX using the HT RNA Reagent Kit (PerkinElmer) prior to construction of libraries. The libraries were constructed with the PerkinElmer Sciclone NGS Workstation using the TruSeq RNA protocol (Illumina). Briefly, mRNA was isolated from 1 μg of total RNA by a poly-A+ pull-down using biotin beads, and fragmented. Following first- and second-strand synthesis, the cDNA fragments were blunt ended, and then their 3′ ends were adenylated to prevent formation of chimeric molecules during the adapter ligation. After ligation of the adapters, the fragments were subjected to a bead-based size selection using Beckman Coulter XP beads (Beckman Coulter). This removed the majority of unligated adapters, as well as any adapters that may have ligated to one another. Prior to hybridization to the flow cell, the cDNA constructs were amplified by PCR with a primer cocktail that annealed to the ends of the adapters to selectively enrich fragments with adapter molecules on both ends. The insert size of the libraries was verified by running an aliquot of the DNA library on a PerkinElmer GX using the High Sensitivity DNA chip (PerkinElmer) and the concentration was determined by using a Qubit dsDNA HS Assay (Life Technologies) and qPCR.

### Sequencing

Libraries were normalized to 15 nM, and cluster generation was carried out on a paired-end flow cell on the Illumina cBot utilizing the TruSeq PE Cluster Kit v3 (Illumina), according to the manufacturer’s instructions; this included a 1% PhiX Control v3 spike (Illumina). The sequencing was carried out on an Illumina HiSeq 2000 using TruSeq SBS v3 Sequencing kit (Illumina), with one library (sample) or three libraries per Illumina flow cell lane (for pupal and larval samples, respectively). Image analysis and basecalling was performed with the Illumina data analysis pipeline HiSeq Control Software v 1.5.15.1, RTA v.13.48, and CASAVA v1.8.2.

### Sequence Quality Control and Transcriptome Assembly

Reads were processed through several open source packages to obtain a final set of high quality data for all subsequent analyses. The FastQC package version 0.10.1 (Babraham Bioinformatics) was used to assess overall run quality and profiles of the reads. Sequencing adapters and primers used in library generation were identified and clipped using the package Trimmomatic version 0.30 (Bolger et al. 2014), with parameters allowing a seed mismatch of up to two bases and adapter matches defined as an alignment score of 10 between the adapter and read. The same package was next used to inspect and remove poor base quality sequences by iteratively trimming bases from the 5′ and 3′ read ends with Phred Quality Scores < 13 (removing base calls with accuracies <95%). Finally, a 4-bp sliding window was used to remove internal bases with Phred quality scores of < 13. Only reads ≥40 bp in length were retained following the trimming steps.

Both paired and unpaired reads were mapped to the *A. gambiae* PEST strain (AgamP4.1) reference genome sequence using Bowtie version 2.1.0 and Tophat version 2.0.10 (Langmead and Salzberg 2012; Kim et al. 2013). Tophat parameters allowed a maximum of six mismatches per read, consisting of single nucleotide polymorphism and/or indels ≤6 bp in length. All mapped reads were assembled de novo into transcript models (i.e., only the genome sequence was used as reference and the existing predicted transcriptome was not provided) using Cufflinks under default parameters (Trapnell et al. 2013).

### Transcript Expression and Analysis

The 12 sets of full length and partial transcript assemblies generated by Cufflinks were processed using Cuffmerge (Trapnell et al. 2013). Those transcript models with exact splicing structure across two or more of the samples were merged to maximize the accuracy of the models and to remove transcript redundancy between the samples. Comparison of the resulting set of merged transcripts with the most recent *A. gambiae* gene build (AgamP4.2) revealed that the latter is highly deficient; nearly 20% of transcripts from this study correspond to new transcribed regions (yet unannotated genes), or regions with an overlap with the annotated genes. Consequently, our transcript set originating from autosomes and the X chromosome served as a reference for the analysis of expression levels. Transcripts mapping within scaffolds that have not been assigned to a chromosome (annotated as UNKN within AgamP4) were excluded from the analysis.

We used two methods to quantify gene expression. In the first, we implemented the Cuffdiff program from the Cufflinks suite to normalize expression of transcript isoforms based on FPKM (fragments per kilobase of exon per million fragments mapped) values, which were scaled based on the geometric means of the libraries (Anders and Huber 2010). Both unique reads and reads that map to multiple transcripts were included in the analysis, with the latter probabilistically assigned to transcripts (Trapnell et al. 2013). In the second method, read counts per gene were calculated using HTSeq (Anders et al. 2015), and using the union mode to handle reads overlapping more than one genomic feature. Only uniquely mapped reads were used to calculate gene expression (supplementary table S1, Supplementary Material online). To enable comparison of gene expression across samples, raw read (fragment) counts were normalized and converted into RPKM expression values. This was performed by dividing the number of uniquely mapped reads per gene by kilobase length of the gene’s total exonic region and then by the total number of uniquely mapped reads (in millions) in the sample. As an alternative to total count normalization, we employed a quantile normalization (75th percentile normalization suggested in Bullard et al. 2010) to reduce the effects of outliers on the RNA-seq data. Finally, we implemented the median normalization (Dillies et al. 2013), but the results were very similar to the 75th percentile normalization (data not shown).

Total exonic length was calculated for each gene by summing up lengths of all nonoverlapping exons using the GenomicFeatures package from Bioconductor. Sequencing metrics are shown in supplementary table S1, Supplementary Material online. Normalized read counts per gene for the larvae and pupae data sets are presented in supplementary tables S2 and S3, Supplementary Material online.

For each expression quantification method we compared the median expression levels from the X chromosome and the autosomes at a range of thresholds of minimum expression levels, between FPKM/RPKM = 0, which includes both active and nonactive genes, and FPKM/RPKM ≥ 40, in which only highly expressed genes were taken into account. We similarly calculated the ratios of median expression from chromosome 3 and chromosome 2 (chr3:2). Furthermore, a comparison between the sexes was obtained by computing the male to female ratios of these values (Method 1). As an alternative male to female comparison, we first calculated the male to female expression ratio for each gene. Then, we took the medians of these ratios for the relevant chromosomes (i.e., X, A, chr2, and chr3), and computed the corresponding X:A or chr3:2 ratios (Method 2). Both methods of male to female expression comparison yielded highly consistent results.

To evaluate significance of differences in expression between chromosomes and the sexes, we computed the confidence intervals for the X:A, chr3:2, and M:F ratios of chromosomal ratios by bootstrapping using the “boot” package in R (Davison and Hinkley 1997; Canty and Ripley 2015). We performed 10,000 bootstrap replicates for each of the above mentioned ratios (with the minimum expression thresholds ranging from 0.2 to 15), stratifying by chromosome and sample. We also performed the bootstrap analysis for genes in each expression-level decile separately (considering the average male and female expression).

A sliding window analysis of distribution of the expressed genes on the X chromosome and of the relative expression magnitude along the X chromosome was conducted using a custom script in R. Numbers of genes expressed above different RPKM thresholds were counted within a 1-Mb window allowed to move in 100-kb steps each time. The same sliding window scheme was employed to calculate the X:A expression ratios using the median expression of the X-linked genes located within each 1-Mb window and the median expression of all the autosomal genes.

To test for underrepresentation of male-biased genes on the X chromosome, first we defined male- or female-biased genes as differentially expressed genes (Cuffdiff *q* value < 0.005) with a fold change above 50% between sexes. Then, we performed a Fisher’s exact test for the independence of male/female bias of a gene from its X/autosomal location. This test reveals differences in the distribution of male- and female-biased genes between the X chromosome and the autosomes, under the null assumption that male- and female-biased genes would be equally distributed between the X chromosome and the autosomes.

To understand if it was appropriate to exclude some larval replicate samples from the analysis, we computed the sum of the squared differences in expression (RPKM) between each of the replicates and the others, and tested for significance by random permutations of expression of each gene across replicates. One male and one female replicate was found significantly different from the other replicates (*P* < 0.001), as apparent also in supplementary fig. S1, Supplementary Material online.

### Testes Sample, RNA Sequencing, and Analysis

Testes were dissected from 2,000 early (up to 4-h old) pupae and from 200 third instar and early fourth instar larvae, and stored in RNA*later* (Ambion) at −20 °C until RNA isolation. RNA was isolated using RNeasy Mini kit (Qiagen), followed by poly-A+ mRNA isolation using Oligotex mRNA Mini kit (Qiagen). The mRNA was treated with TURBO DNase (Ambion) and its integrity evaluated using the Agilent 2100 Bioanalyzer. Then, the mRNA was used to synthesize double-stranded cDNA using the SMART™ PCR cDNA synthesis kit (Clontech) according to the manufacturer’s recommendation. Fragmentation of 1 μg of double-stranded cDNA by nebulization, fragment ends polishing, adaptor ligation, isolation and estimation of the single-stranded template library concentration, emulsion PCR with library beads, loading on the 454 plate, and pyrosequencing on the 454 GS FLX platform (Roche 454 Life Sciences) using Titanium chemistry was performed according to the published protocol (Margulies et al. 2005). Sequence reads were mapped to the AgamP4.1 gene models using GS Reference Mapper v2.3 (Roche). Raw read counts per gene were normalized and converted into RPKM expression values as described above, and are presented in supplementary table S5, Supplementary Material online.

## Results and Discussion

### The Data Set

To explore dosage compensation of the X chromosome in *A. gambiae*, we analyzed transcriptomes of sexed whole-body 12-h-old fourth instar larvae and 4-, 10- and 20-h-old pupae. The larval transcriptomes were collected as three biological replicates, while for the pupae a single sample was taken at each time point. Expression estimates from the same-sex pools of larval replicates were strongly correlated (males: Spearman ρ = 0.83–0.94, females: ρ = 0.86–0.91; *P* < 1.0 × 10^−10^). Pupae samples were similarly correlated despite being collected at different time points (males: ρ = 0.87–0.90, females: ρ = 0.85–0.88; *P* < 1.0 × 10^−10^). However, MA plot comparisons of the samples indicated that the total count normalized data appeared in part severely biased (supplementary figs. S1 and S2, Supplementary Material online). The 75th percentile normalization (Bullard et al. 2010) largely removed the bias within the pupae (supplementary fig. S2, Supplementary Material online); therefore we treated these data as replicates from the pupal developmental stage. However, in two of the larval samples the bias was only slightly decreased (data not shown). Consequently, we analyzed larval data both with the two divergent samples excluded and with all three replicates included (the latter shown in Supplementary Material online).

In total, over 2.7 billion 101-bp-long reads, corresponding to 274.6 Gbp of sequence data, were collected from the 12 samples. Following the read quality filter steps, 99.3% of all reads were retained, with a mean 226.6 million high quality reads per sample (supplementary table S1, Supplementary Material online). After reference-based mapping, a mean 199.7 million reads per sample (88.1%) were mapped to the genome, and of these 184.6 million (92.5% of all mapped reads) aligned uniquely, that is, aligned to only one location within the genome based on the mapping parameters used (see Methods).

Following read assembly, a total of 225,347 transcripts from the 12 transcriptomes were identified within genomic scaffolds of known chromosomal origin. Merging of the transcripts across all the samples reduced the above number to a set of 46,247 nonredundant transcripts within 16,510 autosomal and X-linked genes. All gene expression analysis was undertaken on this common, and as full length as possible, set of transcript models. The nonredundant transcript set was compared with the *A. gambiae* transcripts within the Agam4.1 gene build, which holds 15,478 transcripts within 13,638 genes. We found that only 45.1% of the transcripts from our set match exactly the intron structure within the Agam4.1 transcripts. A further 36.8% of transcripts share at least one splice junction with the Agam4.1 transcripts, and so are potential novel isoforms. The vast majority of the remaining 18.1% of transcripts fall either within intergenic regions (14.1%) or are exonic overlaps with the Agam4.1 transcripts. Detailed characterization of the larval and pupal transcriptomes is beyond the scope of this study and will be presented elsewhere.

### Dosage Compensation Analysis

Tests for dosage compensation involve either comparisons of gene expression levels between the X chromosome and the autosomes within each sex, between males and females for the X and the autosomes, or—in a phylogenetic framework—comparisons of expression levels of the X-linked genes and their autosomal orthologs in an outgroup, as a proxy for the ancestral genes on the nascent sex chromosomes. Each method has its own limitations and assumptions that may not necessarily be tenable. Taking into account the posited autosomal origin of sex chromosomes and the X upregulation in males hypothesized to have emerged as a counter balance to a gene loss from the Y, the appropriate test for dosage compensation seems a comparison of the average X expression with the average autosomal expression for males and females separately (Vicoso and Bachtrog 2011; Walters and Hardcastle 2011; Harrison et al. 2012). However, because nonhomologous genes of various functions are compared, their chromosome-wide average expression levels may not be equal even if dosage compensation operates. In the male to female expression comparisons, sex-biased genes may confound the conclusions (Nozawa et al. 2014). That strategy also carries an implicit, potentially invalid, assumption that the female X chromosomes have the same expression levels as their autosomal ancestors. In addition, although this strategy would allow detection of the X chromosome-wide regulation of expression, it provides no indication on the direction of the regulation (i.e., hypertranscription in males or reduction of transcription in females). Finally, the strategy based on contrasting the expression levels of orthologs from the ingroup and the outgroup assumes that the gene expression levels are static for long evolutionary times. This assumption may not be met, especially for more distant outgroups (Nozawa et al. 2014), and is likely more problematic for taxa with high rates of interchromosomal gene movement, such as insects. Similar results obtained from alternative strategies should strengthen the conclusions regarding presence and extent, or absence, of dosage compensation. Therefore, to test for dosage compensation in this study, we calculated, separately and in combination, the X:A and male to female ratios of gene expression in *A. gambiae* (no appropriate data for the outgroup comparisons were available to us).

Different criteria used to select genes for computing X:A ratios can profoundly influence the outcome of the dosage compensation analysis (Kharchenko et al. 2011; Jue et al. 2013). Arguably, only transcriptionally active genes should be used for the analysis. Genes not active in a given tissue or a developmental stage are irrelevant to the question of dosage compensation. More importantly, their inclusion lowers the estimates of overall transcriptional output from the chromosomes and, if unequally distributed between the X chromosome and the autosomes, nonactive genes may bias the X:A ratios (Kharchenko et al. 2011). However, distinguishing transcriptionally active genes expressed at very low levels from the nonactive genes is not straightforward. Background transcriptional signal (Struhl 2007) and experimental artifacts, such as sequencing errors, erroneous read mapping to nonactive paralogs, or trace amounts of genomic DNA contaminating the sequenced mRNA samples, can contribute to the spurious association of reads to nonactive genes. The probability of sampling functionally irrelevant reads increases with greater sequencing depths (such as in the case of this study), but so does the chance of detection of rare transcripts. Therefore, rather than using an arbitrary filtering cut-off (a specific number of reads to regard genes as active), we explored the X:A expression ratios at a range of thresholds of minimum transcription levels. This approach allowed us to get an insight into compensation of genes expressed at increasing levels. Numbers of genes included in each data set corresponding to the individual thresholds of minimum expression are presented in supplementary table S6, Supplementary Material online.

Natural variability in the median expression levels is known to exist among chromosomes. For example, average magnitude of transcription from human autosomes 10 and 11 substantially differs from the average transcription levels of all the other autosomes (Kharchenko et al. 2011). The significance of X:A values should be considered in the context of such a variability, and to do so, we contrasted the X:A ratios to the ratios of median expression from chromosomes 3 and 2. These chromosomes represent the only two pairs of autosomes in the *A. gambiae* karyotype and they have roughly comparable numbers of genes (chr2: 8,570, chr3: 7,598). Thus, if we consider as true that levels of expression are balanced across the genome, the average expression magnitudes for chromosomes 2 and 3 should be approximately equal. In line with this expectation, in the HTSeq-calculated data set, the chr3:2 estimates are consistently close to unity in males and females at both developmental stages and across the whole range of the minimal FPKM/RPKM thresholds (supplementary fig. S3, Supplementary Material online). This is not the case with the cuffdiff-calculated data set, in which the chr3:2 ratios are below or around 0.8 at low expression thresholds, and gradually approach 1.0 as the thresholds increase. Thus, the HTSeq-based method appears to more accurately estimate chromosome-wide levels of expression in *A. gambiae*. Consequently, our conclusions about dosage compensation are based on the gene-centric HTSeq-calculated data set. However, the conclusions of our study would not be substantially different, if drawn from the cuffdiff-based expression quantification.

**Fig. 1.—:**
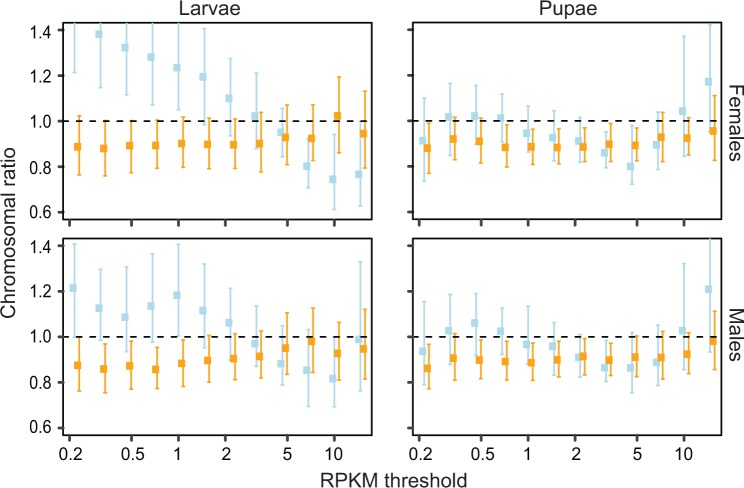
Analysis of chromosome-wide expression ratios as a function of increasing thresholds of minimum expression (RPKM) levels. The ratios of median expression (HTSeq-based RPKM) from the X chromosome and the autosomes (X:A, blue) are shown along with the ratios of expression from chromosome 3 and chromosome 2 (chr3:2, orange). For each threshold, dots represent the median and bars indicate the 95% confidence intervals from bootstrap. Only values for thresholds between 0.2 and 15 RPKM are presented, because for thresholds outside this range the confidence intervals are meaninglessly large.

In pupae, the X:A ratios are very close to 1.0 and within the chr3:2 ratio ranges (fig. 1 and supplementary figs. S3 and S4*A*, Supplementary Material online), consistent with complete compensation. In larvae, the X:A values oscillate between the high of 1.5 and the low of 0.7, depending on the RPKM threshold. Although similar trend is evident in both sexes, in females the X:A confidence intervals exceed 1.0 and fall outside of the chr3:2 range when genes with low RPKM (≤1.0) are included, indicative of overexpression from the female X chromosomes. As the expression thresholds increase (between RPKM 6.0 and 10.0), the X:A ratios drop below 1.0, but are still within the ranges of the chr3:2 confidence intervals. Such fluctuating X:A values, if taken out of the chr3:2 ratio context, could lead to different interpretations of the data, depending on the expression thresholds applied. This observation becomes relevant in light of a recent study of dosage compensation based on human proteomic data (Chen and Zhang 2015). Because of the low sensitivity of mass spectrometry, only the most highly expressed proteins were used in the analysis, from which the authors concluded that there is no X upregulation at the protein level. Although their conclusion may be correct, our results indicate that analyses based on arbitrarily selected fraction of the data should be interpreted with considerable caution. In contrast to the patterns observed in our study, an inverse pattern (with low ratios of expression from the sex chromosomes vs. the autosomes at low FPKM thresholds, and the ratios increasing to 1 with the increasing expression levels) was found in a moth, *Manduca sexta* (Smith et al. 2014), and in humans (Kharchenko et al. 2011). Moreover, the X:A ratios are invariably close to 1.0 in *A. stephensi* adult males and females across RPKM thresholds ranging from 0 to 4 (Jiang et al. 2015).

Our genome-wide comparisons revealed substantial differences between the two developmental stages in the expression variance, especially of X-linked genes. The high variance remains even after excluding the divergent larval samples (data not shown). Because pupae samples were sequenced to a three times greater depth, we tested how difference in the number of analyzed reads affects the observed expression patterns. We used FastqSampler from the ShortRead package in R to resample the reads from the pupal stages to the depth level of the larvae, and computed chromosomal ratios for the pseudoreplicates (supplementary fig. S5, Supplementary Material online). Apart from a slightly increased expression variance, the plots were practically identical to the ones for pupae in supplementary fig. S3, Supplementary Material online.

**Fig. 2.—:**
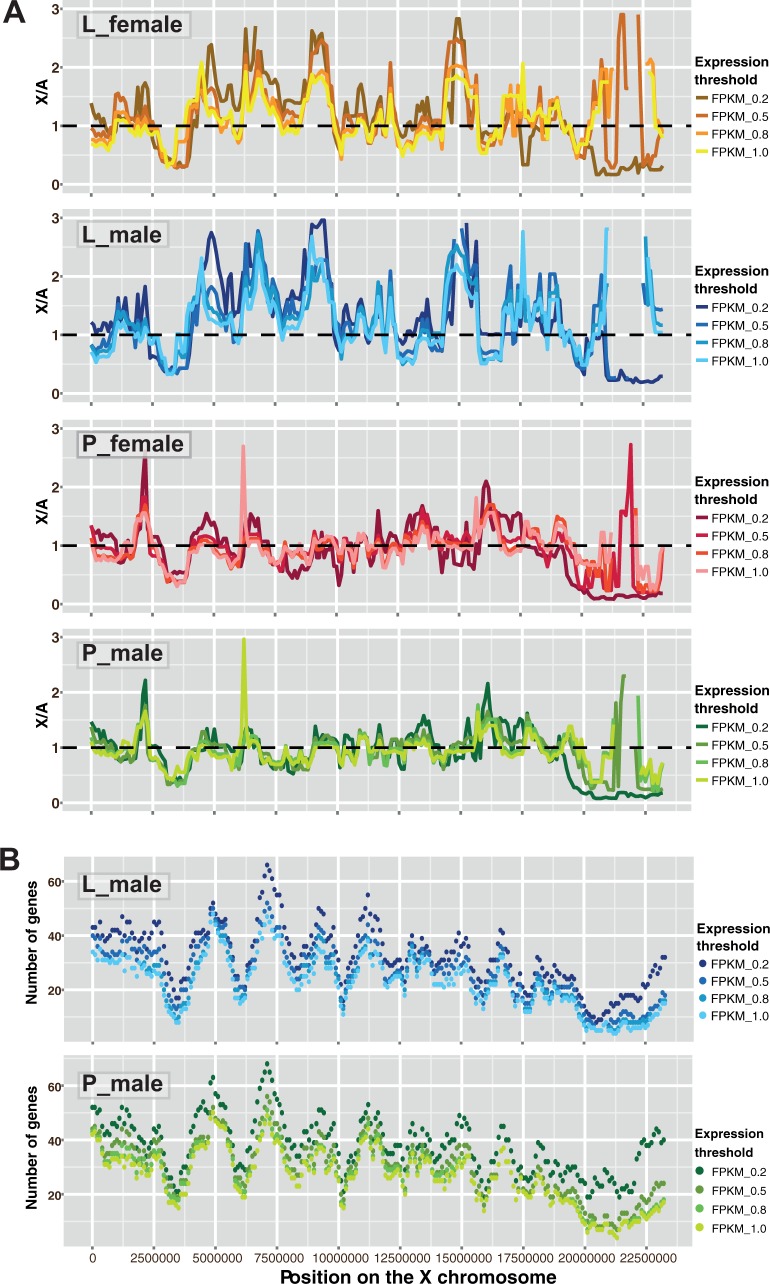
An analysis of the relative (X:A) expression magnitude (*A*) and the density of genes expressed above five RPKM thresholds (*B*) along the X chromosome. The X:A ratios correspond to the values of median expression of the X-linked genes (located within a 1-Mb window sliding in 100-kb steps) divided by the overall median expression of autosomal genes. Calculations are based on average values from medians of three samples. The sliding window analysis was performed for genes expressed above five minimum RPKM thresholds, with the highest RPKM threshold of 1.0. At the higher thresholds (not shown), there were not enough expressed genes in a number of windows, making the analysis meaningless, because the X:A ratios strongly deviated from 1.0 in those regions. The lines in *A* are interrupted in regions, in which the X:A ratios are beyond the adopted scale.

A sliding window analysis reveals differences in the expression magnitudes between larvae and pupae in a detailed pattern along the X chromosome (fig. 2). In pupae, median expression of the X-linked genes encompassed in a sliding 1-Mb window and of all the autosomal genes is roughly balanced across most of the X chromosome’s length. Stronger departures of the X:A ratios from unity are, in general, restricted to the few X chromosome regions apparently prone to biases, because each of these regions are occupied by a smaller than average number of genes, which are expressed at either low or high levels (Pearson’s *r* = −0.464, *P* = 7.7 × 10^−14^; supplementary fig. S6, Supplementary Material online). In contrast, most of the X chromosome in larvae is hyperexpressed relative to the autosomes, with the strongest hyperexpression in the regions with high gene density.

In summary, despite some variation in the data, our comparisons of the X:A expression ratios support complete compensation in male pupae. These conclusions are in a general agreement with an earlier study suggesting that the male X-linked genes are compensated in the *A. gambiae* adult somatic tissues (Baker and Russell 2011) and with the analyses of dosage compensation in *A. stephensi* adults (Jiang et al. 2015). Similarly, dosage compensation through upregulation of the male X chromosome has been found in representatives of six other nondrosophilid dipteran families (Vicoso and Bachtrog 2015). The patterns of the X:A ratios in the fourth instar larvae are more complex and dependent on the expression level cutoffs, but also oscillate around 1.0.

**Fig. 3.—:**
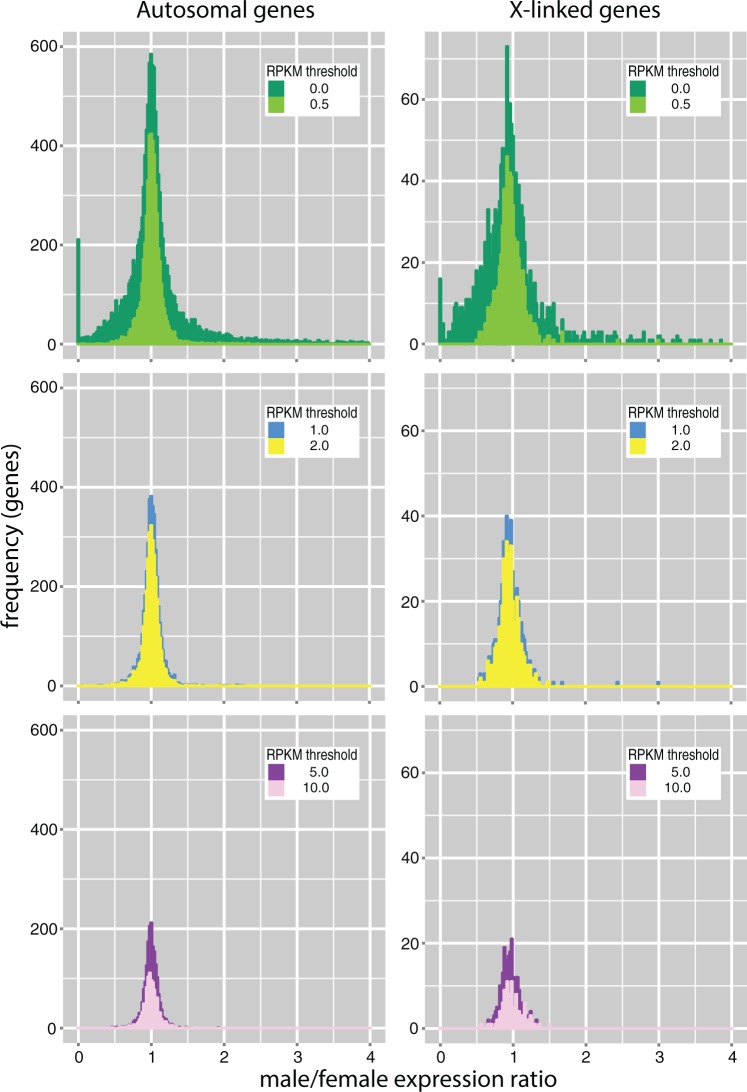
Ratio distributions of X-linked and autosomal genes from male pupae compared with those from female pupae. Ratios from each of the three time points were averaged to generate the plots. The data are presented for genes at six thresholds of minimum expression (RPKM) levels (0, 0.5, 1.0, 2.0, 5.0, and 10.0). The ratio of 1.0 indicates no expression difference between the sexes.

**Fig. 4.—:**
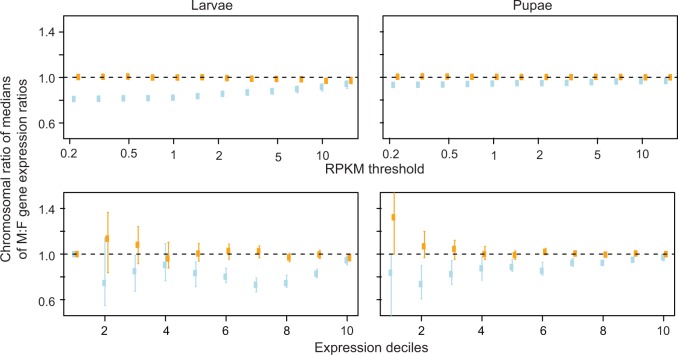
Comparison of chromosome-wide male-to-female expression ratios as a function of increasing thresholds of minimum expression and of expression deciles. The plots show the values computed using Method 2 (cf. Materials and Methods). For expression deciles plot, genes were divided into deciles based on expression level, with the first decile corresponding to genes with the lowest expression. The ratios of median male-to-female expression ratios from the X chromosome versus autosomes (blue) are shown along with the ratios from chromosome 3 versus 2 (orange). Values lower than 1 for the X:A ratio mean that genes on the X chromosome tends to be less male biased compared with genes on the autosomes. For each point, dots indicate the median and bars indicate the 95% confidence intervals from bootstrap.

**Fig. 5.—:**
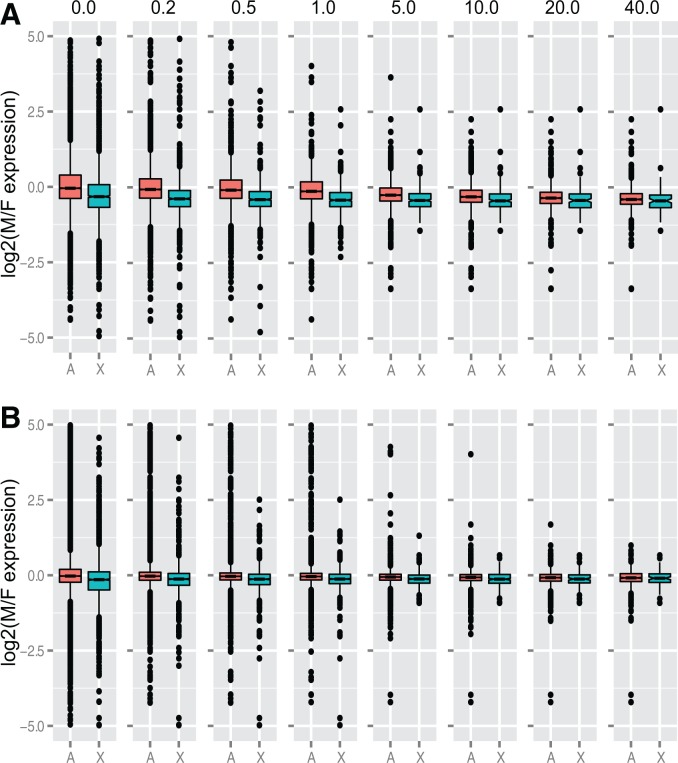
Log_2_ of the male to female ratios of autosomal and X chromosome expression in larvae (*A*) and pupae (*B*) at increasing thresholds of minimum expression (RPKM) levels. Numbers above the plots indicate the minimum RPKM thresholds.

The comparisons of gene expression between males and females show similar results. The ratios of chromosome-wide (median) expression are close to 1.0 in males and females regardless of the developmental stage, the method of gene expression quantification, or expression level threshold, although a minimal dosage effect seems to be present in males (larvae X_male_:X_female_ 0.89–1.08, A_male_:A_female_ 0.89–0.99; pupae X_male_:X_female_ 0. 92–0.96, A_male_:A_female_ 0.97–1.01; supplementary tables S2 and S3, Supplementary Material online). We found virtually the same patterns at the gene level, when a male to female ratio distribution analysis was conducted for all expressed genes in pupae, with the ratios plotted in bins of 0.02 increments (fig. 3). This type of analysis provides strong power to determine trends of gene expression, because numerous data points (ratios for different genes) are present in the major bins (Sun, Johnson, et al. 2013). Although a number of genes located on the autosomes and the X chromosome are sex biased to various extents, the majority of genes on the autosomes are approximately equally expressed in both sexes (peak around the value of 1.0), and on the X chromosome only slightly female biased (fig. 3 and supplementary fig. S7, Supplementary Material online). The X chromosome bias is strongest when weakly expressed genes are included, and decreases with increasing expression level thresholds. However, it persists at all thresholds analyzed. Comparison of chromosome-wide male-to-female expression ratios revealed stronger female bias in the expression of the X-linked genes in larvae than in pupae (figs. 4 and 5; supplementary figs. S8 and S4*B* and *C*, Supplementary Material online). In pupae, the bias was driven mainly by genes expressed at low levels, while in larvae by relatively highly expressed genes (cf. Expression deciles in fig. 4). The female bias could result from mild dosage effects, a deficit of male-biased genes on the X chromosome, or a combination of the two. In an attempt to disentangle these alternatives, we performed a genome-wide analysis of differential gene expression. We found that the male-biased genes are significantly underrepresented on the X in the pupae data set (Fisher’s exact test, *P* < 0.0001; supplementary table S4, Supplementary Material online), consistent with findings in adult *A. gambiae* (Baker et al. 2011; Magnusson et al. 2012; but see Hahn and Lanzaro 2005, who did not detect significant deviation from an equal distribution of male-biased genes, apparently artifactually, due to limitations of their experimental design and suboptimal genome annotation used for the analysis). Thus, the deficit of the X-linked male-biased genes appears to be an important factor contributing to the observed female bias in pupae. In contrast, the bias appears to be driven by dosage effects in larvae, in which we found no indication of underrepresentation of the X-linked male-biased genes.

Incomplete compensation was reported for *D. melanogaster* late larvae and prepupae based on comparisons of the male X to autosomal expression and on the analysis of mean expression of X-linked genes in males and females (Perry et al. 2014; Sun, Johnson, et al. 2013). In addition, data from *Drosophila pseudoobscura* suggested that compensation was closer to complete in larvae and to a lesser extent in pupae, than in adults (Nozawa et al. 2014). Irrespective of the compensation effects, the above studies indicate that in *Drosophila* dosage compensation levels vary and are developmental stage dependent.

Is there no dosage compensation in the *A. gambiae* testes? We attempted to answer that question using the Roche 454 pyrosequencing-generated transcriptomic data from testes of early pupae. Although germline and somatic cells build the testes, the large majority of the pupal testes volume is composed of the germline cells, including large, highly transcriptionally active primary spermatocytes (testis in *Anopheles* is a thin-walled sack, composed of somatic cells, and filled with cysts of germline cells; cf. figure 3 in Magnusson et al. 2012), and the bulk of mRNA originates from the germline. Thus, our pyrosequencing data correspond primarily to the germline expression. Because of the nature of the sequencing platform, the number of collected reads is very small (950,938 in total, of those 755,053 left after trimming of poor quality reads and 434,957 uniquely mapped to AgamP4 gene sequences located on the autosomes and the X chromosomes) and, thus, is not strictly quantitative compared with the data for whole larvae and pupae; still, it provides an instructive insight into and a rough estimate of expression in testis. The chr3:2 and the X:A median expression ratios at the RPKM threshold of 1.0 are 0.95 and 0.44, respectively, which could indicate absence of dosage compensation, and which is in accordance with an earlier study (Baker and Russell 2011). Alternatively, but not mutually exclusive, low expression from the X may also be due to the X demasculinization, resulting from X inactivation during male meiosis or from selection for accumulation of female-beneficial genes (Magnusson et al. 2012; Meiklejohn and Presgraves 2012). In our data the fraction of genes expressed from the X chromosome is almost twice as low in the testis transcriptome as in the transcriptomes of whole larvae or pupae (6.7%, 12.9%, and 11.1% for testis, larvae, and pupae, respectively). Some caveats should be applied to this testes data set owing to the lower transcriptome coverage, relative to the Illumina data sets, which most likely has resulted in an undersampling of gene expression. For this reason, we did not use this data set to build our gene models, and only a global, chromosome-wide expression ratio was calculated, with the expectation that the effect of undersampling is reduced by averaging across all genes. If the X:A ratio estimate was found comparable with the above (i. e., to be close to 0.5) in a similar, but higher coverage RNA-seq experiment, it would indicate lack of compensation in the male germline, rather than a result of X inactivation during meiosis (Baker and Russell 2011), because in the testes of the *A. gambiae* early pupae the great majority of germline cells are premeiotic. Components of dosage compensation machinery driving the X chromosome hypertranscription in somatic cells are not expressed in the *Drosophila* male germline (Rastelli and Kuroda 1998). Consistent with that finding, the X in the germline cells appears to be not compensated (Meiklejohn and Presgraves 2012). Similar to *Drosophila*, in two other higher dipteran species, *Themira minor* (Sepsidae) and *Ephydra hians* (Ephydridae), the X chromosome is not upregulated in testes (Vicoso and Bachtrog 2015). Data from our study and a previous report (Baker and Russell 2011) on *Anopheles* (a lower dipteran) strengthen the notion that this phenomenon may be a general feature in Diptera (Vicoso and Bachtrog 2015).

### Candidate Dosage-Sensitive Genes

**Fig. 6.—:**
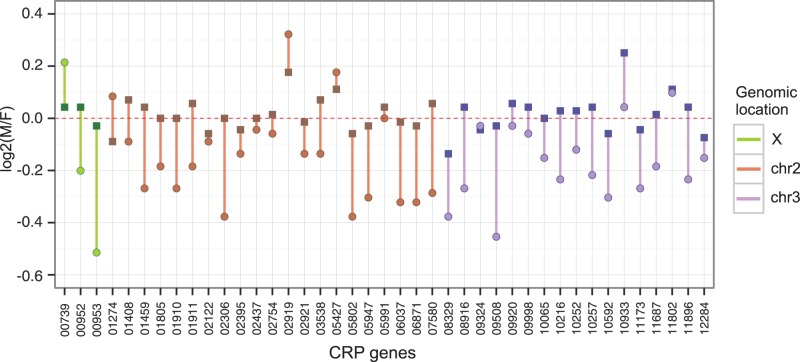
Ratios of male to female expression of candidate haplolethal and haplosterile CRP genes in the 4 h (squares) and 20 h (circles) old pupae. For clarity, the data points for each gene are connected with a line. Line color indicates linkage of genes to chromosomes.

The X chromosome dosage compensation must have evolved to prevent deleterious effects of reduction in transcription of dosage-sensitive (haploinsufficient) X-linked genes during the evolution of the XY chromosomal system (Ohno 1967; Pessia et al. 2012). However, it would be incorrect to assume that all X-linked genes equally transcribed in males and females are dosage sensitive. A detailed screen of chromosomal deletions in *Drosophila* revealed surprisingly few haploinsufficient genes not only on the X chromosome, but across the whole genome (Cook et al. 2012). In total, 49 genes or short genomic regions (of those, only 11 X-linked) were found to be haplolethal or haplosterile, and additional 56 (including 7 X-linked) genes to have lesser phenotypic effects. The large majority of these genes encode cytoplasmic ribosomal proteins (CRP). The CRP genes have been reported as haploinsufficient in a wide range of organisms, including yeast, zebrafish, and humans (Kim et al. 2010) (and references in Marygold et al. 2007); therefore, their haploinsufficiency status most likely holds for mosquitoes. By definition, the haploinsufficient X-linked genes are expected to be expressed at equal levels in both sexes and, as such, they should constitute an optimal control group to evaluate the extent of natural sex-dependent expression variation in dosage compensated genes. In this context, we took a closer look at the transcription of haplolethal/haplosterile CRP orthologs in *A. gambiae* pupae. Although there are only three X-linked genes in this category, their analysis reveals more general trends pertinent also to the autosomal CRP genes. Their male to female expression ratios are not necessarily very close to 1.0 as might be expected (average range from 0.88 to 1.02 for X-linked and 0.88 to 1.12 for autosomal genes). Ranges of the male to female CRP expression ratios markedly widen with the age of pupae (fig. 6; supplementary fig. S9 and table S6, Supplementary Material online), which suggests a tighter control of the CRP expression levels in the early (4 h old) than in the late (20 h old) pupae. Thus, there can be a considerable variation of expression levels even in the apparent dosage-sensitive genes. There is a slight, but significant, shift toward female bias in expression of all CRP genes in the late pupae compared with the early ones (Mann–Whitney *U* test, *P* < 0.00001). Moreover, the male to female ratios of CRP gene expression drops, on average, 2-fold with age. A plausible interpretation of the above observations is that the requirement for maintaining stoichiometry of the ribosome constituents is more stringent when the demand for protein synthesis machinery is particularly high. The most dramatic metamorphosis processes, including imaginal appendage formation and a massive restructuring of the thoracic and head musculature, require extensive protein synthesis and occur largely in earlier *A. gambiae* pupae (Clements 1992). At the age of 20 h, pupae approach eclosion, most metamorphosis processes come to an end and the pharate adults are nearly fully formed; however, the development of female pupae is protracted by several hours (as a rule, females emerge later than males). Thus, the differences in the pupal development rates may contribute to the female bias in the CRP expression in late pupae.

### Dosage Compensation Machinery

In *D. melanogaster* dosage compensation is epigenetically controlled and tightly linked to the sex determination pathway. Although the actual compensation mechanisms may be more complicated, it is widely accepted that hypertranscription of the male X chromosome is mediated by the MSL (Male-Specific Lethal) complex, which binds exclusively to the X and consists of at least five proteins and two noncoding RNAs (Gelbart and Kuroda 2009; Philip and Stenberg 2013; Sun, Fernandez, et al. 2013). The complex is not assembled on the female X chromosomes, because translation of a critical subunit, *msl2*, is repressed by SXL, a female-specific protein operating at the top of the sex determination pathway (Bashaw and Baker 1997). Mutations of the MSL complex genes lead to misregulation of this intricate machinery, which, depending on genetic lesions, is highly deleterious or lethal to either males or females. A link between the sex determination and dosage compensation control exists also in *C. elegans* (Miller et al. 1988) and apparently is common to other taxa, including *Anopheles.* However, the mechanisms controlling compensation in *Anopheles* must be different from those in *Drosophila*, because the *sxl* homolog is neither involved in sex determination in mosquitoes nor are its transcripts sex specific (Saccone et al. 2002). It is unclear whether the components of the MSL complex are involved in maintaining dosage compensation in *Anopheles*. Even though orthologs of all five genes encoding MSL proteins are present in the analyzed transcriptomes, two genes (*msl1*, which remains unannotated in *A. gambiae*, and *msl2*) are extremely diverged (11–14% amino acid identity and 31–33% similarity to *Drosophila* proteins; supplementary figs. S10 and S11, Supplementary Material online), which may indicate their altered functions. In another lower dipteran, *Sciara ocellaris*, immunostaining analyses revealed that orthologs of *Drosophila* MSL complex proteins do not associate exclusively with the X chromosome, but bind equally the X chromosome and the autosomes, indicating that different proteins implement dosage compensation in these two fly species (Ruiz et al. 2000). It is likely that interfering with expression of the components of the mosquito dosage compensation system would lead to similar detrimental phenotypic effects as in *Drosophila*, and result in sex ratio distortion. Therefore, shedding light on the composition of the dosage compensation machinery in *Anopheles* would not only advance our knowledge of fundamental processes that shape male and female phenotypes, but may also augment the repertoire of genetic tools applicable to mosquito control through population suppression (Alphey 2014).

## Supplementary Material

Supplementary tables S1–S7 and figures S1–S11 are available at *Genome Biology and Evolution online* (http://www.gbe.oxfordjournals.org/).

Supplementary Data
