# Supplementary material for: Dosage Compensation in the African Malaria Mosquito Anopheles gambiae
Source: Genome Biol Evol. 2016 Jan 18;8(2):411–25. doi: 10.1093/gbe/evw004 (PMC4779611; doi:10.1093/gbe/evw004)
Supplement: Supplementary Data [file supp_8_2_411__index.html]

Dosage Compensation in the African Malaria Mosquito Anopheles gambiae — Supplementary Data 

# Dosage Compensation in the African Malaria Mosquito *Anopheles gambiae*

## Supplementary Data

files

- Supplementary Data - xlsx file
- Supplementary Data - pdf file
