# Supplementary material for: Dosage Compensation in the African Malaria Mosquito Anopheles gambiae
Source: Genome Biol Evol. 2016 Jan 18;8(2):411–25. doi: 10.1093/gbe/evw004 (PMC4779611; doi:10.1093/gbe/evw004)
Supplement: Supplementary Data [file supp_evw004_Supplementary_Material.pdf]

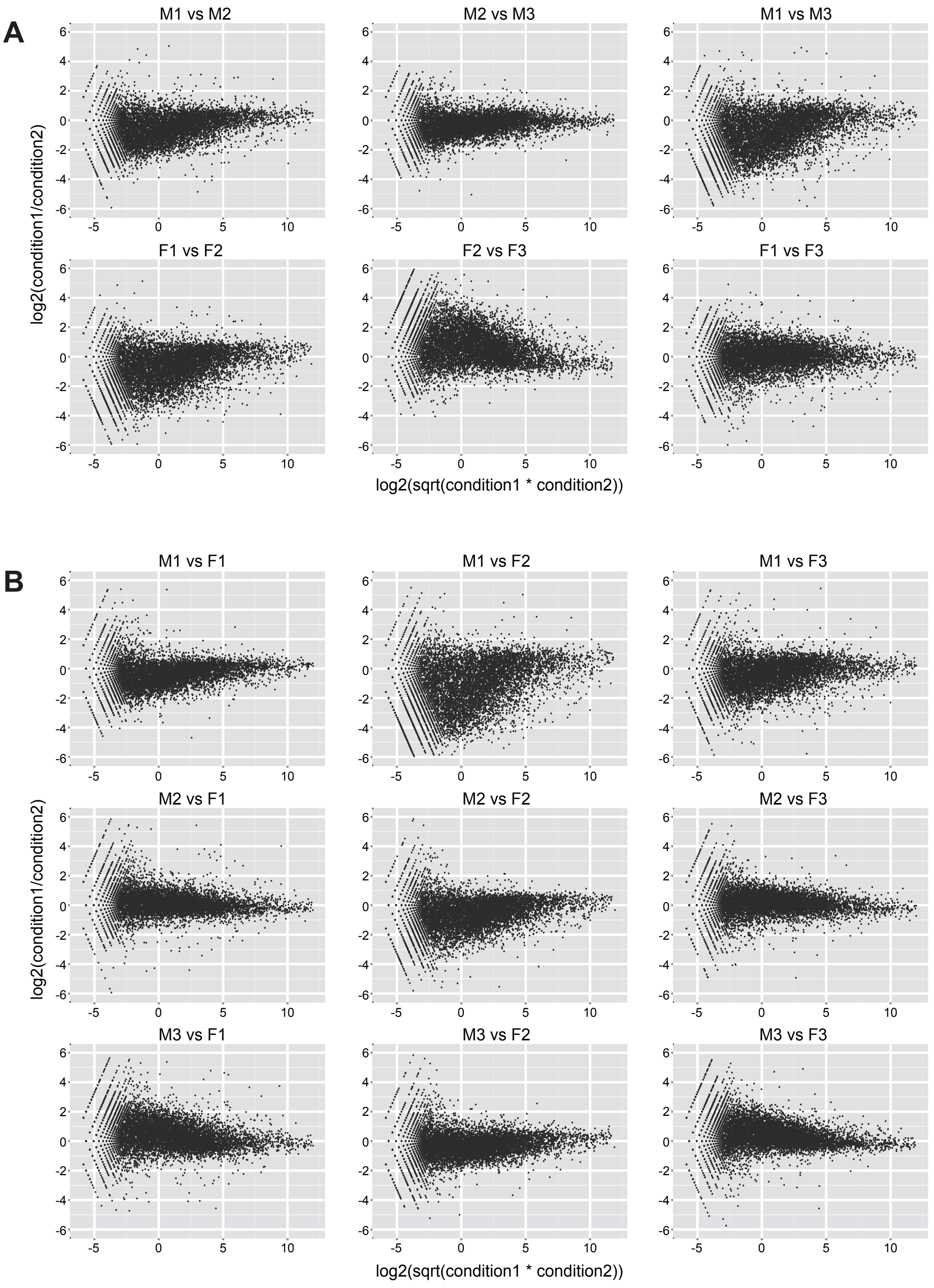

Fig. S1. MA plots showing within-sex (A) and between-sex (B) comparisons of global gene expression in the larval samples. The total count-normalised data shown in the plots indicate that samples M1 and F2 are strongly biased. Other normalization methods used did not substantially correct the bias (not shown).

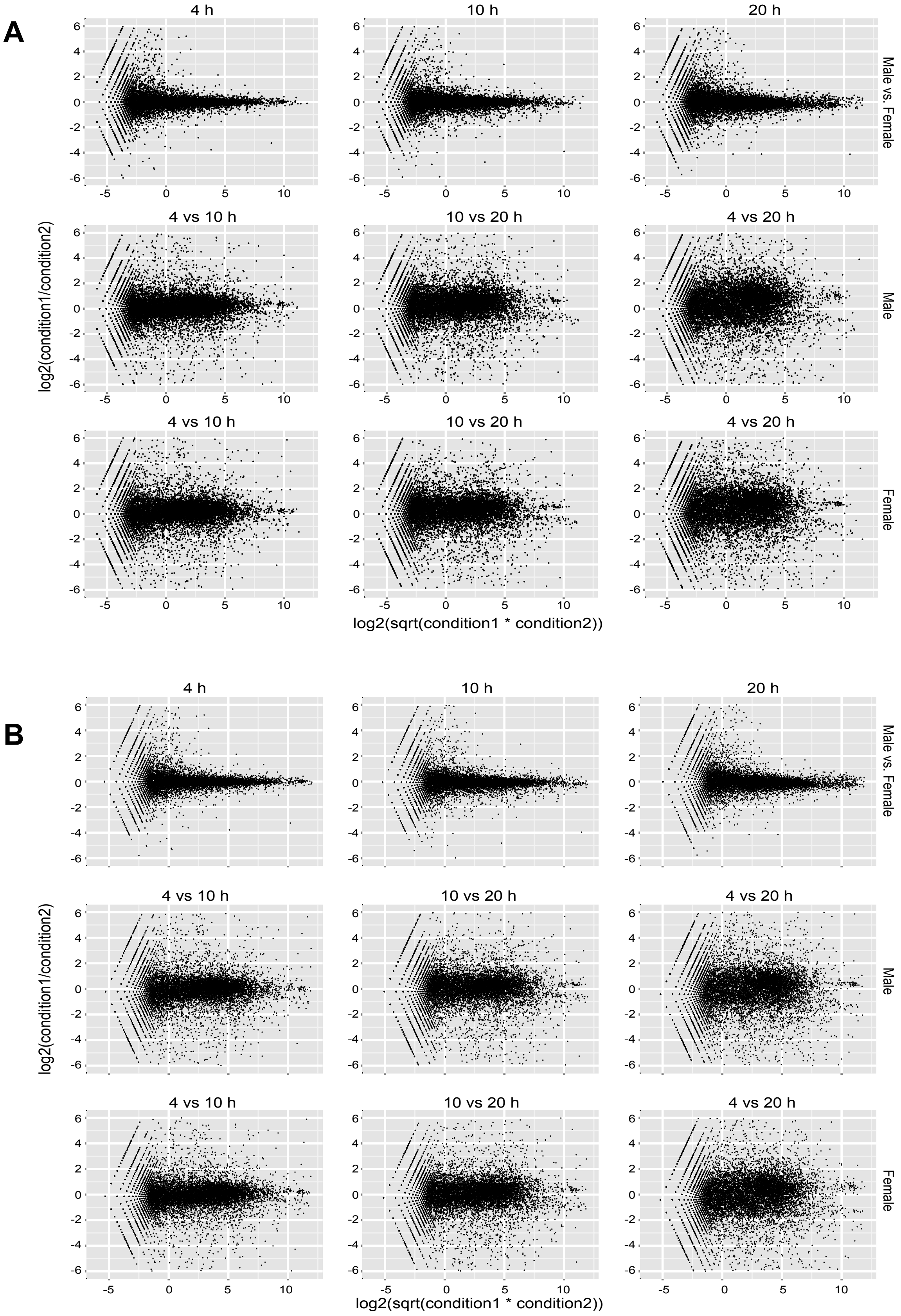

Fig. S2. MA plots showing within-sex comparisons of gene expression in the pupae samples. The total count-normalised data (A) indicate global shift towards lower expression in 20 h samples relative to earlier timepoints. This phenomenon is due to a relatively small number of genes with heavily biased expression between early and late pupae. Application of the 75th percentile-normalization (B) largely eliminated the bias.

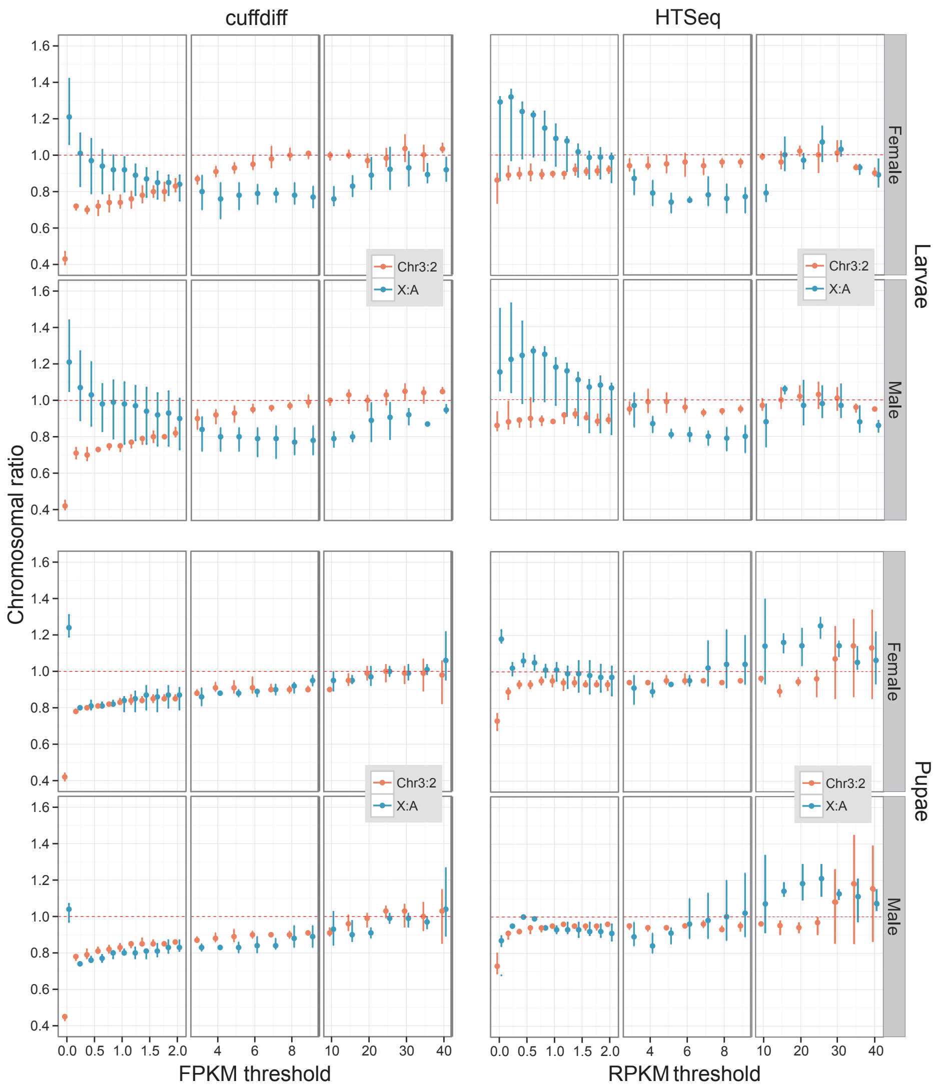

Fig. S3. Analysis of chromosome-wide expression ratios as a function of increasing thresholds of minimum expression (FPKM/RPKM) levels. The ratios of median expression from the X chromosome and the autosomes are shown along with the ratios of expression from chromosome 3 and chromosome 2. For each threshold, the ratios from three replicates are shown as the minimum, the maximum, and the intermediate value. The data are presented for Cufflinks- and HTSeq-based (total-count) methods of gene expression quantification.

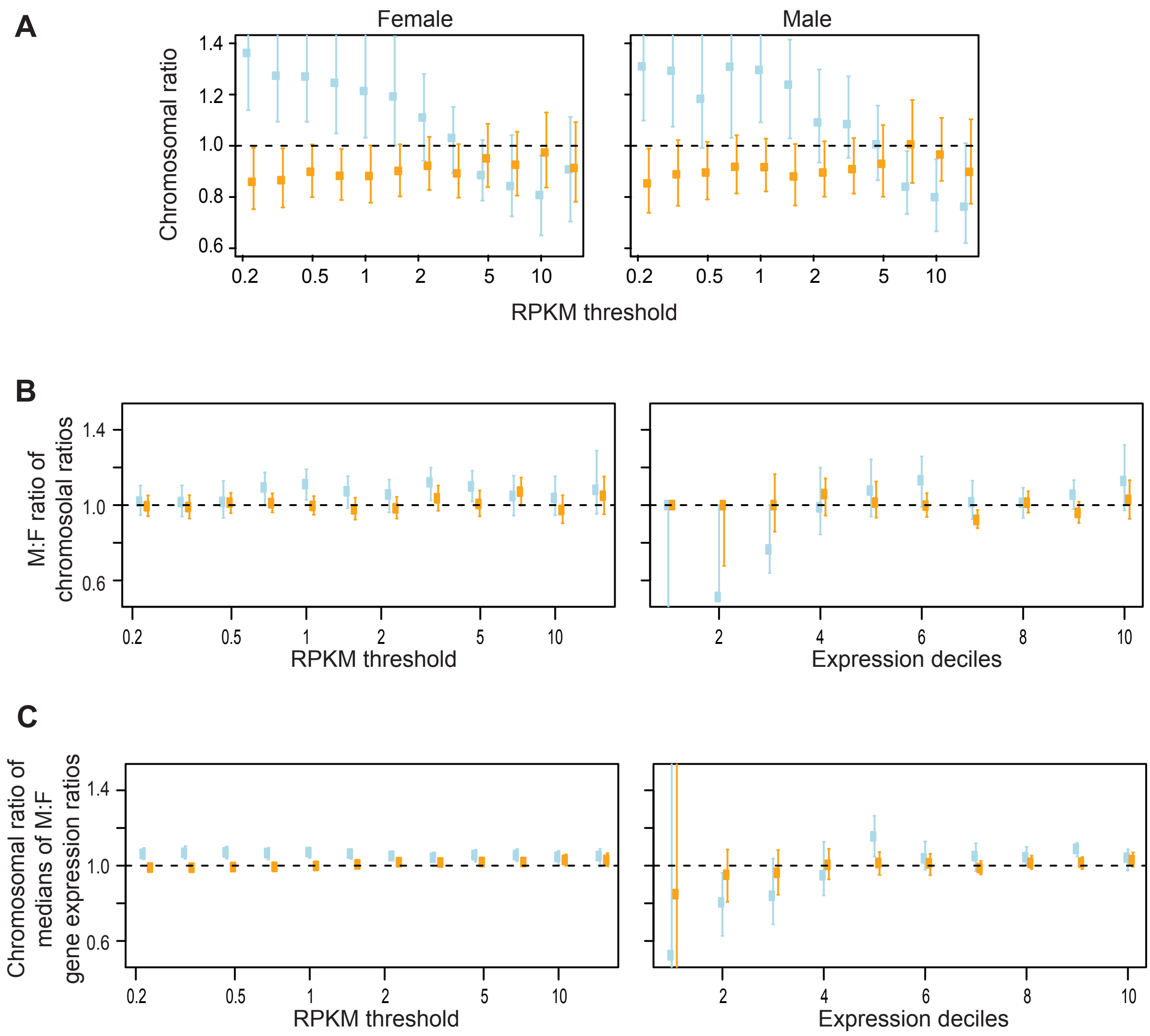

Fig. S4. Analysis of chromosome-wide gene expression in 12 h old 4th instar larvae (all three replicates). Inclusion of the divergent replicates resulted, in general, in smoothing the female bias. (A) Analysis of chromosome-wide expression ratios as a function of increasing thresholds of minimum expression (RPKM) levels; cf. fig. 1. (B and C) Comparison of chromosome-wide male-to-female expression ratios as a function of increasing thresholds of minimum expression and of expression deciles; cf fig. 4.

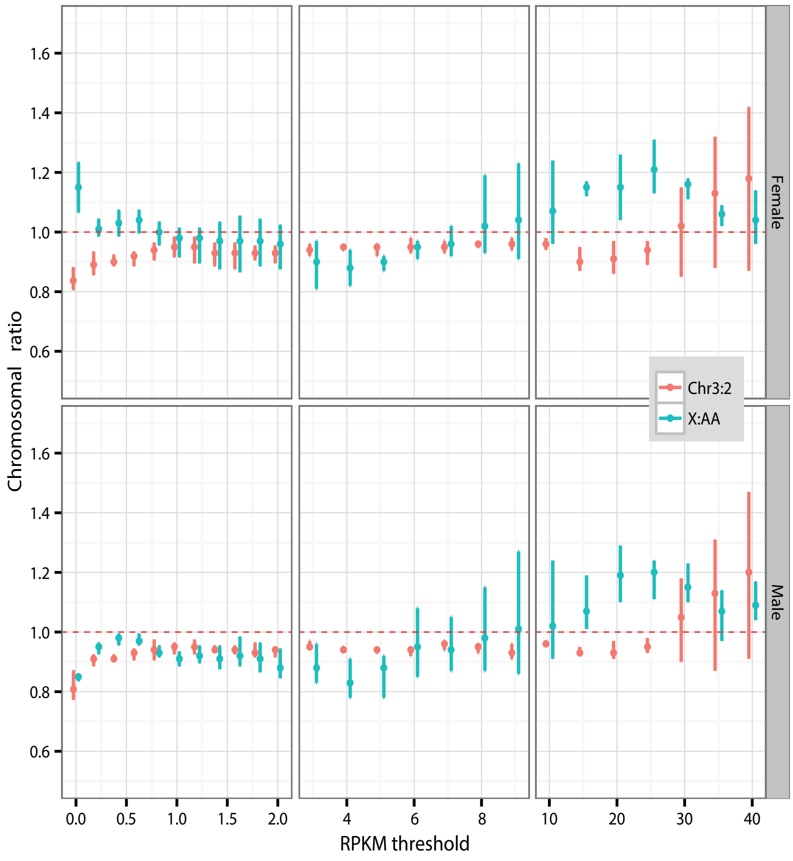

Fig. S5. Analysis of chromosome-wide expression ratios as a function of increasing thresholds of minimum expression (RPKM) levels in pseudoreplicates from pupae. One pseudoreplicate was sampled from each of the pupae timepoints. The ratios of median expression from the X chromosome and the autosomes are shown along with the ratios of expression from chromosome 3 and chromosome 2. For each threshold, the ratios from three pseudoreplicates are shown as the minimum, the maximum and the intermediate value.

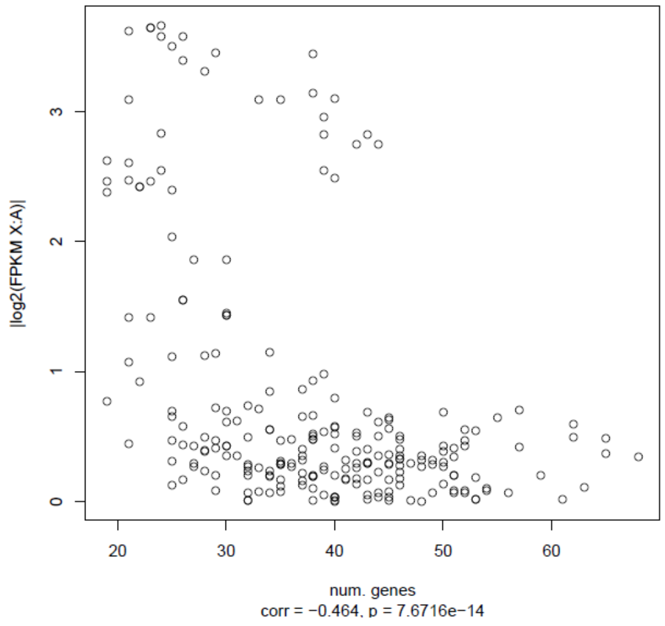

Fig. S6. Test for association between expression levels (absolute values of log2(X:A)) and a number of genes expressed at the minimum FPKM level of 0.2 from the X chromosome in male pupae. The data set used to generate the plot was used to generate part of Fig. 2.

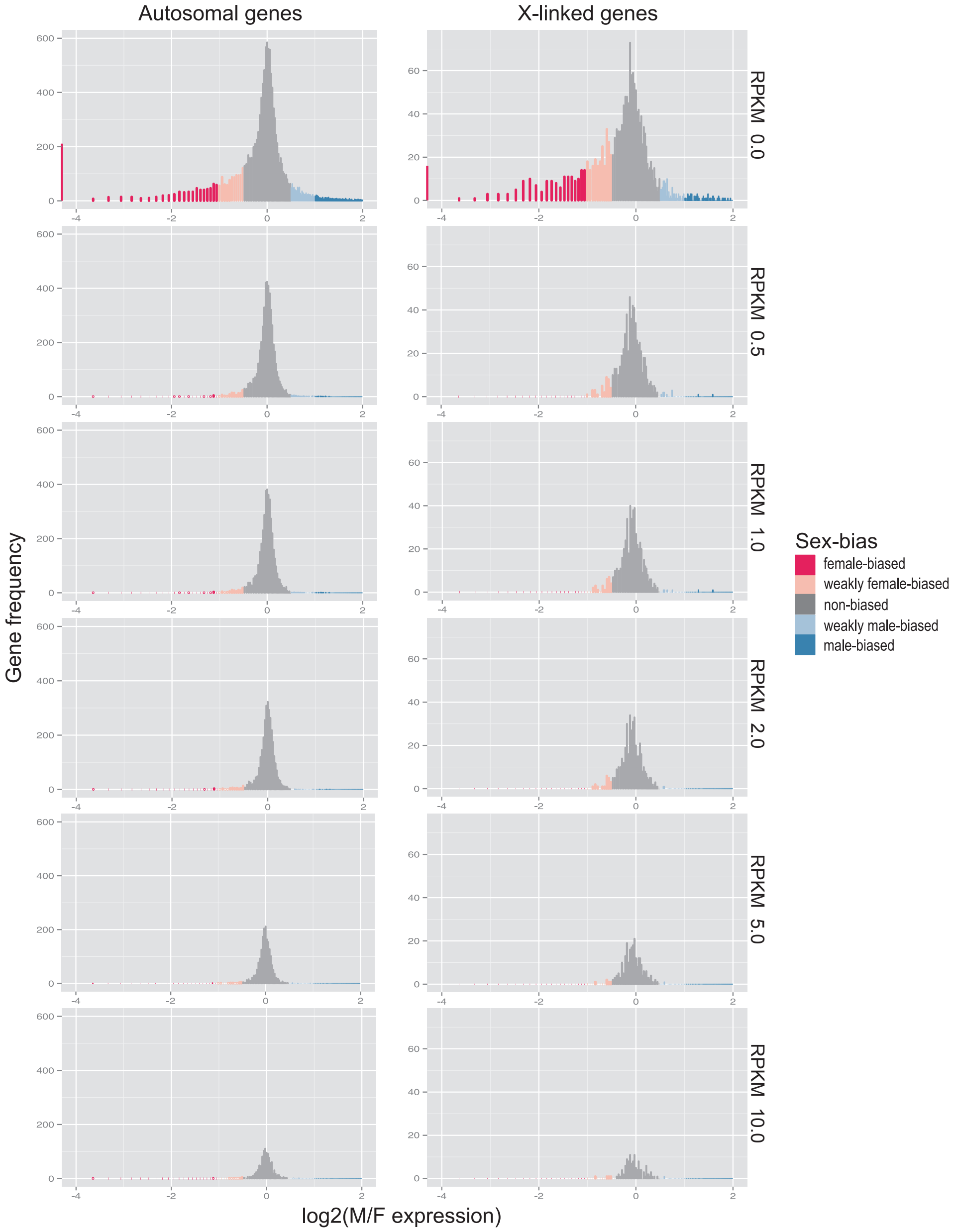

Fig. S7. Ratio distributions of X-linked and autosomal genes from males compared with those from females. Ratios from each of the three pupal timepoints were averaged to generate the plots. The data are presented for genes at six thresholds of minimum expression. The ratio of 1.0 indicates no expression difference between the sexes. For each of the thresholds the sex bias is colour-coded. Genes with two-fold or greater expression bias are regarded as sex-biased, and those with fold expression bias between 1.5 and 2, as weakly sex-biased.

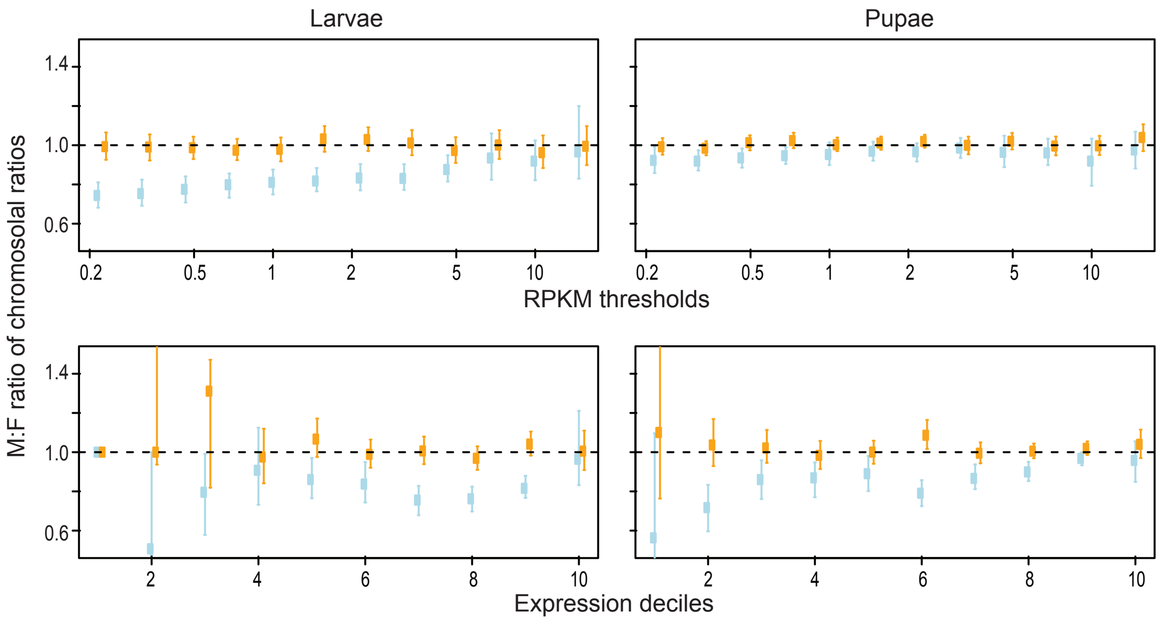

Fig. S8. Comparison of chromosome-wide male-to-female expression ratios as a function of increasing thresholds of minimum expression and of expression deciles. The plots present the values computed using method 1 (cf. Materials and Methods). For expression deciles plot, genes were divided into deciles based on expression level, with the first decile corresponding to genes with the lowest expression. The ratios of median male-to-female expression ratios from the X chromosome versus autosomes (blue) are shown along with the ratios from chromosome 3 versus 2 (orange). Values lower than 1 for the X:A ratio mean that genes on the X chromosome tends to be less male-biased compared to genes on the autosomes. For each point, dots indicate the median and bars indicate the 95% confidence intervals from bootstrap.

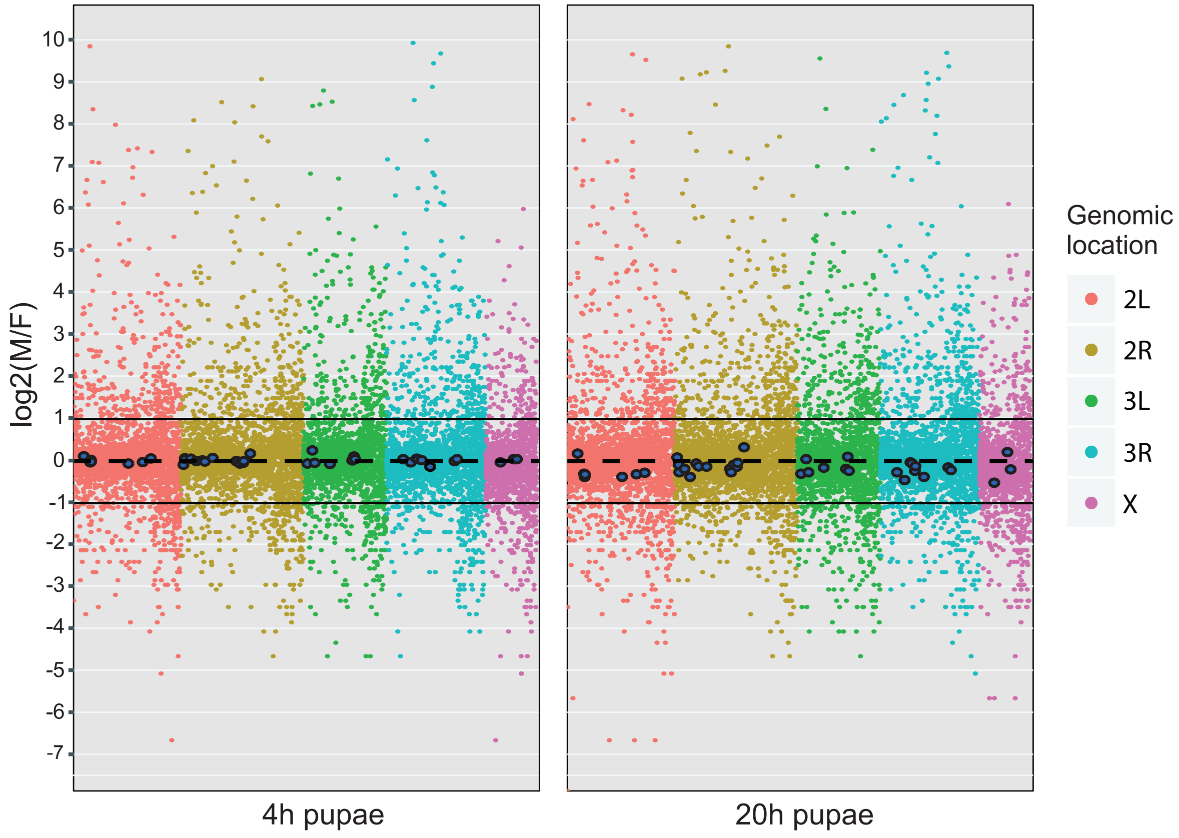

Fig. S9. Ratios of male to female expression of candidate haplolethal and haplosterile cytoplasmic ribosomal protein genes (large blue dots) in the 4 h and 20 h old pupae in the context of chromosome-wide expression patterns.

Fig. S10. CLUSTAL X-generated amino-acid alignment of the MSL1 protein from *D. melanogaster* and of its ortholog from *An. gambiae*.

```
Agam_MSL1       MGASSASAINMMDTMDDHVYCNSTTATVAATSPSTTVT-VTTSATATAQTTVAVAAASPR
Dmel_MSL1       -----MDKRFKWPPKKRANYLESPYPHIPSRGRQRNLHGHPNQTQHLHQHPGKIYERQQY
                      .      . .   * :*. . :.: . . .:   ...:    * .  :   .

Agam_MSL1       AVPKPSVRESSTSDDQYQQSSPSQYSPSPSSPLPSSPQPASPRPSSPVPSSSVPSSPAPS
Dmel_MSL1       GNGRGGHGGGNNNYRKLLHSLPAEHGGGAMAPPSSGGTVCAGADMVKLISENNNLRRMVM
                .  : .   ....  :  :* *:::. .. :* .*.   .:      : *..

Agam_MSL1       SPVPSMEDETLMMEDDETEPDPP----PLYGREEQSNESQTEGEEAAGENEAEAGFVNDV
Dmel_MSL1       LNLNLMQEQTDSIAAKDKELDDQSAKMSVVKAQNEELKQAVAQLEAANQELCKQLRRKNQ
                  :  *:::*  :  .:.* *      .:   :::. :. .   ***.:: .:    ::

Agam_MSL1       YDYGSQSGNDERLVNPDDS-----EYDEEDGDDDDEEDEDAEDLDNIPSDSGVVTDDQQG
Dmel_MSL1       RRNDNDDDDDDPPLPPAAPQQKLIRCHAETQTVFREREQGTQTIDAQPQFANALPRGINM
                   ..:..:*:  : *  .     . . *      *.::.:: :*  *. :..:. . :

Agam_MSL1       DDPGDDNNGSGMAEALVAMN----------EVKQLQSILLLHLDLIQEQGDQILNKDKMI
Dmel_MSL1       KESPALDHHAGAVTNQPASKRSESKGRGEFNGKKVSTFILQRMNQDFEHHIHEQTEVAEE
                .:.   :: :* .    *  :          : *::.:::* :::   *:  :  .:

Agam_MSL1       IRLKDENEVLKHQLELANRRVSVMMMQLQQNGLALPGDVQQQLVMQQPAPKQEGTVSPLT
Dmel_MSL1       HEMEAHKEQISQEEDQLVAEEDHLHMQEVHTEEVVGGDIFHDALESIEMEVVTEELVDME
                 .:: .:* :.:: :    . . : **  :.  .: **: :: : .         :  :

Agam_MSL1       IRSQMENGRMRTIILKSSSSPPFVSSSVADGGPLFGAPCLPIRNPEVSSTVSPAKEELLD
Dmel_MSL1       EHGQSVDANG---HIEEDDDEDDEDDENSDKDDDSEEDDYPWMHSDADVNARTEEELWQN
                 :.*  :..     ::....    ... :* .        *  :.:.. .. . :*   :

Agam_MSL1       EIVLQNDPHQSPFYNVDDDEEDEDGEENNGEDEEDFRTDNNEVDGEIFLNYSKSDDDESL
Dmel_MSL1       QNYLLELDPTEEKTCAPSAHSTPNHQQKSSTQAEIRKEGNQNRITEKLLQLKPEPMVDAL
                :  * :    .    . . ..  : :::.. : *  : .*::   * :*: . .   ::*

Agam_MSL1       SPPMN-----HDYEDQQQQQQQQQQQQQDEEHDSDDVQVVNDLYLQQQEQQQQQQQQQYM
Dmel_MSL1       EAPILPKWVAFKKKDKEHESVPESPEVPKQQPHQEDAIVDHNAIKNQLEVPKPDLKPKDQ
                ..*:      .. :*::::.  :. :  .:: ..:*. * ::   :* *  : : : :

Agam_MSL1       LLQQQQQQQQQQQHTLQEHFVGMKTEEMDEEEEEG---------GYQSNGMAGEEDEEE
Dmel_MSL1       PKDEQRQDGQLDVRVEPQEDVRKVQKETLKRQPEDAPKHLPKAVAPKVTKTSSRESTLPK
                  ::*:*: * : :   **..  ::.* :..: *:.            ::  : *.   :

Agam_MSL1       EDEEDEGATSPAQYVSDDPANYDDTPMVMIDDGAVEVTMETDSPESQQSSQDSRDSQDSQ
Dmel_MSL1       ANTADIKDAPAQKVIANHQSTKTQTDPVKTQRLQVKIRQYEMHPDMRTGSSAPSDIRKQK
                 :  *   :.. : :::. :.  :*  *  :   *::      *: : .*. . * :..:

Agam_MSL1       DSQESQSYRQESQDSSQQGQPEVNVPFVESSTVGGEAATHQHQQPVDDDCKEVIADGKAE
Dmel_MSL1       NVDPVSTPETKTIKSKSMLVNDKKTTSETSQSPDQEIDVETVRRKLAEHLKKELLSQSHS
                : :  .: . :: .*..    : :..   *.: . *  ..  :: : :. *: : . . .

Agam_MSL1       IKREQTAESSQVS------------ARGDIKPKGALERMPYAGCSREAPTEGGGENGREG
Dmel_MSL1       SQVTLKKIRERVATNLIYPPPSAPVSSTTITPAPTPSTTPTPGSTPQHAVTSSMDQEISA
                 :   .   .:*:            :   *.*  : .  * .*.: : .. .. ::  ..

Agam_MSL1       VGRSRGGNARMPRQFNPTLHCPSGNSSDGSVAARRNCKPTAAYMVTQKQYVSGSWKDDAV
Dmel_MSL1       AKSKSKAAEQIATPLTPQSNSSVSSTTSTIRKTLNNCSPHTYSKATARSGKLQSRFRTAT
                .  .  .  ::.  :.*  :.. ..::.    : .**.* :   .* :.    *    *.

Agam_MSL1       TAEIEKLLSNEAAELEIPSWTVIEDDGDDPAGGCDPPGGGEEEPSVSASEPSRENISDEA
Dmel_MSL1       FPYSTRTWEDQEFHCDNEFFLEEADELLADNPSLEIPKWRDVPVPPSSDKIDTELLSDAT
                 .   :  .::  . :   :    *:      . : *   :   . *:.: . * :** :

Agam_MSL1       YAKRHTKLEIDERRRKKWDVQRIREQKHIERLKKRQLKEQPVEQEAQKAITTLYPTVDTL
Dmel_MSL1       FERRHQKYVKDEVDRKCRDARYMKEQIRLEQLRMRRNQDEVLVALDPLRASTFYPLPEDI
                : :** *   **  **  *.: ::** ::*:*: *: ::: :        :*:**  : :

Agam_MSL1       KYVLVTDDVPVQAFGELIPLLPTNGGFSLPWNQPKPGPSFANPQTSCSTTTSSASTASAG
Dmel_MSL1       EAIQFVNEVTVQAFGENVVNMEARDDFGVPWVDAIEAP------TSIARSKALAEPVATL
                : : ..::*.****** :  : :...*.:** :.  .*      ** : :.: *...::

Agam_MSL1       MGHHGPGPSGLGLLLETKTKFIHRLAPSLQAQKQRFTKRIKKDP
Dmel_MSL1       ASKKIPTTAAEARHQENHSSYVFPKRRKRQKNR-----------
                 .:: * .:. .   *.::.::.    . * ::
```

Fig. S11. CLUSTAL X-generated amino-acid alignment of the MSL2 protein from *D. melanogaster* and of its ortholog from *An. gambiae*.

```
Agam_MSL2         MNPTSLYITTSRLVFQGDIVAG---LQDLNRLLPYLRKSLSCAVCCKLLVEPHSPVTGDC
Dmel_MSL2         -MAQTAYLKVTRIAMRSASNLSKRRVEELNSGLGELRQLLSCVVCCQLLVDPYSPKGKRC
                    . : *:..:*:.::.     .   :::**  *  **: ***.***:***:*:**    *

Agam_MSL2         QHHICRVCVGKHKKLKPACRFCKG---LLQYRENTQLRLLLQCYKSICTFIRTRPVYADI
Dmel_MSL2         QHNVCRLCLRGKKHLFPSCTQCEGCSDFKTYEENRMMAAQLLCYKTLCVHLLHSALFGEL
                  **::**:*:  :*:* *:*  *:*   :  *.**  :    * ***::*..:   .::.::

Agam_MSL2         CKCAGGAGQPREGGGPGPSSAASPHNTATTSPNSLMELIEEGAAFVDDFKCNSGLSKSAY
Dmel_MSL2         A------------GMRPQVARELVPRIKLPPKTTQEFIREGSNISDTF-----------
                  .             *  *. * .     . .*::  *:*.**: : * *

Agam_MSL2         SILPCVFPPPVVTVQQQPAAAEPKPPQGSSAPQVLLEHAQDSFQKQLELAQQRQQQIAQE
Dmel_MSL2         DIFLPQPDLPFLKDMPTSLPAETPPTSAVTTPELPYDHHLNISDIEAEAAATAEQ-----
                  .*:      *.:.    . .**. *... ::*::  :*  :  : : * *   :*

Agam_MSL2         RQRLQALLPHQRLPPSPSVAAGKLVGQQQQQQTRMAAFPPGVGGSAAKPLNTSSIIRNVL
Dmel_MSL2         ----------GHFSPLPLLPTGSRMGMLSHAGQIVIATESSESGFMDQAWTDQVDLSGTV
                            ::.* * :.:*. :*  .:    : *  .. .*   :. . .  : ..:

Agam_MSL2         PARIGPTIIHAESLAGRTGTTSASPLVPQHVVKNSCVPTTPRIVNITTPQEIKFEAAPTI
Dmel_MSL2         SVSKYTNSGNNFAVSYVMPTSATTKFDPQELQIGQVVQMA---------DSTQLAVLAAV
                  ..   ..  :  :::    *:::: : **.:  .. *  :         :. :: . .::

Agam_MSL2         KTVSSGSTMYSVLYAESGNKFTIKRKPDTVSAAATAAKSVHGGAQQQQQQTTTTTKLATM
Dmel_MSL2         EETVETSTQLTVLSTTVEETVETSTQLEVLTSAEEPNEISDQLANLQVEESDEALVEETV
                  : . . **  :** :    :..   . : :.:::*  . :  .  *: * :::  :    *:

Agam_MSL2         SPLGGAARSTLIPAKAAAATEFEQPRMAMAQKQTAAPNNPIVLQFRPSQARTIQLPLQNQ
Dmel_MSL2         EEAEGTSIPSEVVAEHMEEDQHLDVHTSQSPTQTEMEEAVEEHVATKTQLGHVQTELQDA
                  .   *:: .: : *:     :. : : : : .**    :         :*   :*  **:

Agam_MSL2         QQQQQQHGTVLAKQQTSSSPLQVQMVQQQQQYQLQQNQTVVSLAPQQQQNFRSLIIQQQQ
Dmel_MSL2         ESLQKDFEDAKAAAEEAK---EKEKDLHAISAELQKEDSDEPTLKRKRTRTLKASQAAKI
                  :. *::.   . *  : :.    : :    :  . :**::::  .    ::: .  .      :

Agam_MSL2         QQQQQTATQQTQPSAQSSGQGSQQQQQQQQALKRKGCRCGNATPTPGKLTCCGQRCPCYV
Dmel_MSL2         EPVPSEVKTKVQSGKGALRRIRGKDKEEKVKPPKPKCRCGISGSSNTLTTCRNSRCPCYK
                  :   . .. :.*..  :  :   :::::    :  **** : .:     ** ..*****

Agam_MSL2         DSKSCVDCKCRGCRNPHRADGLKIRRSLSELLFEQYNAPATTATATATVDTLSAGTTASS
Dmel_MSL2         SYNSCAGCHCVCCKNPHKEDYVESDEDDD---LEDFEMPKDVPEPMTQSEEPVVAEPRQE
                  . :**..*:*  *:***: * ::  .. .    :*::: *  .. . :  :   .. . ..

Agam_MSL2         TLTLGPTITPVTYSISALKAASAATPGSSATAVATGTTKVKVISAGQHHQILQTTAKAPP
Dmel_MSL2         ENSMAPPDSSAPISLVPLNN----------------------LQQSQHPLVLVQNEKGEY
                    ::.*. :... *: .*:                         :. .**  :*  . *.

Agam_MSL2         APSVVKTGAGGGRTRTNGGGVGVVGLLPTSKNPPTVQPSSSSSGGGGSTITIRPFVSGGG
Dmel_MSL2         QGFNIFQGSKPLDPVTVG------------------------------FTIRVQLQHTD
                      :  *:    . * *                              :***  :.  .

Agam_MSL2         SFASGSGTTTTTISYLPTNVTSGGAAPTTSSAYGSQYLQSSAATVRLSKPGPSRTSTVTS
Dmel_MSL2         GFGSLPQYAYIMPTIDPPNPPAPSLSPPPPPAPDREVIEPPAKKFRTSRTRRGRANFSAL
                  .*.* .  :    :  *.* .: . :*....* . : ::..* ..* *:.  .*:.  :

Agam_MSL2         GTLGGHPPPATSFGGGGTYAGGKPDALLSQSSVAMMPVVAMPPAGGASFSTPTGVWSPSL
Dmel_MSL2         DTVDELVSGGSRSNSAAGDRSSATDNAHSLFEEIMSGSDDL-------------------
                  .*:.   . .:  ....   .. .*   *  .  *     :

Agam_MSL2         PSNLFGGGGSVASTGTSSPVGGGGGVGGGGSATGSSTAPELSSLSGYDMNSFVTIGHMLD
Dmel_MSL2         ------------------------------------------------------------


Agam_MSL2         PEGVGVIDLCDDLDLNAANLISID
Dmel_MSL2         ------------------------
```
